# Transmission of viral pathogens in a social network of university students: the eX-FLU study

**DOI:** 10.1017/S0950268820001806

**Published:** 2020-08-14

**Authors:** P.N. Zivich, M.C. Eisenberg, A.S. Monto, A. Uzicanin, R. S. Baric, T. P. Sheahan, J. J. Rainey, H. Gao, A. E. Aiello

**Affiliations:** 1 Department of Epidemiology, University of North Carolina at Chapel Hill, Chapel Hill, NC, USA; 2 Carolina Population Center, University of North Carolina, Chapel Hill, NC, USA; 3 Department of Epidemiology, University of Michigan, Ann Arbor, MI, USA; 4 Division of Global Migration and Quarantine, Centers for Disease Control and Prevention, Atlanta, GA, USA; 5 Department of Microbiology and Immunology, University of North Carolina at Chapel Hill, Chapel Hill, NC, USA; 6 Division of Global Health Protection, Centers for Disease Control and Prevention, Atlanta, GA, USA

**Keywords:** Acute respiratory infection, coronavirus, influenza, transmission, university

## Abstract

Previous research on respiratory infection transmission among university students has primarily focused on influenza. In this study, we explore potential transmission events for multiple respiratory pathogens in a social contact network of university students. University students residing in on-campus housing (*n* = 590) were followed for the development of influenza-like illness for 10-weeks during the 2012–13 influenza season. A contact network was built using weekly self-reported contacts, class schedules, and housing information. We considered a transmission event to have occurred if students were positive for the same pathogen and had a network connection within a 14-day period. Transmitters were individuals who had onset date prior to their infected social contact. Throat and nasal samples were analysed for multiple viruses by RT-PCR. Five viruses were involved in 18 transmission events (influenza A, parainfluenza virus 3, rhinovirus, coronavirus NL63, respiratory syncytial virus). Transmitters had higher numbers of co-infections (67%). Identified transmission events had contacts reported in small classes (33%), dormitory common areas (22%) and dormitory rooms (17%). These results suggest that targeting person-to-person interactions, through measures such as isolation and quarantine, could reduce transmission of respiratory infections on campus.

## Introduction

A disease transmission network is a collection of individuals and their connections, where connections correspond to potential infectious disease transmission events. Understanding the pattern of contacts between people has led to a better understanding of transmission dynamics. The utility of networks for understanding infection transmission has been demonstrated in both empirical data [1] and through simulations [2]. The SARS-CoV-2 global pandemic has further highlighted the importance of considering network structures to prevent transmission with proposed intervention on network structures [3]; and applied interventions, such as stay-at-home orders [4], closing non-essential businesses [4] and school/university closings [5].

Due to population density, co-housing, and complex social contact networks; universities are a common setting for infectious disease transmission [6, 7]. Furthermore, universities may act as entry and dissemination points for outbreaks, as observed for novel influenza viruses [8], including the 2009 H1N1 pandemic [7] and conjunctival *Streptococcus pneumoniae* outbreaks [9]. Previous studies on respiratory infection transmission in universities have focused on novel influenza strains [6–8, 10–12], with reliance on self-reported symptoms for case definitions [7, 8, 10, 11]. Without testing for the causative agent of influenza-like illness symptoms through molecular methods, putative transmission events may not reflect the actual pathogen of interest [13]. Work utilizing testing for other seasonal pathogens has focused on aetiology [14, 15]. Contacts in previous work have been broadly defined through geographical locations [11, 12, 16] or organisation membership [7]. A more detailed approach, one better able to capture the complexity of social contacts in university settings, is needed.

Previous work on respiratory infections among university students using network data has focused on network diversity and subsequent infection risk [17, 18]. These studies have suggested that individuals with more diverse networks are at greater risk of upper respiratory tract infection, possibly due to the increased chance of exposure to respiratory infections. However, network diversity measures captured contacts incapable of transmission (e.g. phone calls, social media, etc.); and putative transmission events were not described. Other work has focused on describing networks of contacts capable of respiratory transmission, without identification of transmission events [19].

We conducted a large social contact network study of students living on a university campus to describe the putative transmission of viral pathogens between students. As seen with recent respiratory virus epidemics and pandemics (e.g. SARS, MERS, SARS-CoV-2, etc.), the study of transmission events needs to be extended beyond influenza A. Our work adds to the existing research by ascertaining a wider range of seasonal pathogens and usage of a novel network approach that includes identification of contacts. The design of this study allowed for the identification of asymptomatic transmission and transmission events occurring in a wider variety of settings than previous studies.

## Methods

### Study design

Within the eX-FLU cluster randomised trial (NCT01472536), 590 students living in dormitories at the University of Michigan were followed for 10 weeks during the 2013 influenza season [20]. Data are available upon request through a data use agreement. Briefly, students were recruited through in-person informational tables, a study website, and chain referral sampling. Enrolled participants were asked to nominate eligible social contacts, and asked to complete the same enrolment and social contact nomination. To be included, students had to be at least 18 years old and living in one of six selected dormitories (chosen based on representativeness of on-campus students). Students were grouped into clusters based on residence and randomised to either the 3-day isolation arm or the control arm. From 19 January 2013 to 5 April 2013 (excluding March 2–8 for winter break), students were asked to report any influenza-like illness symptoms. The case definition for influenza-like illness was self-reported coughing in addition to one of the following symptoms: fever, feverishness, chills or body aches. Influenza-like illness cases randomised to 3-day intervention were asked to self-isolate for 3 days in their dormitory room. Control individuals were not asked to modify their normal behaviours. Students were able to report influenza-like illness symptom onset anytime during the study through email, phone, a web-based reporting system or the weekly follow-up survey. Influenza-like illness cases had specimens collected at day 0, day 3 and day 6 post-symptom onset. Healthy contacts who reported social contact with an influenza-like illness case in the most recent weekly survey were also asked to contribute specimens for testing. Under this specimen collection algorithm, asymptomatic infections could be captured as intermediary links in transmission chains of the same pathogen of the influenza-like illness case or capture asymptomatic infections of different pathogens. For healthy contacts with a positive pathogen test, their onset date was assumed to be the date of the first positive sample.

Specimen collection and testing has been previously described elsewhere [20, 21]. Briefly, a nasal sample was collected from a single naris by a double-headed swab. A second double-headed swab was used to collect a throat sample. Specimens were mixed and tested for influenza A/B, human metapneumovirus (hMPV), rhinovirus, parainfluenza 1/2/3, adenovirus, respiratory syncytial virus (RSV) and human coronavirus (HCoV) 229E/OC43/NL63/HKU1 via quantitative PCR. Details on pathogen incidence and symptomology have been reported elsewhere [21].

### Participant information

Over the 10 weeks, participants were asked each week to report face-to-face contact with other study participants, their relationship to the person and where the contact occurred for the prior 7 days. In total, 83% (*n* = 379) completed more than half of the weekly surveys [20]. Locations of contacts were restricted to locations within 14 days of potential transmission events. Students were considered ‘important contacts’ if either selected the other as one of the top three people they had contact with. We further supplemented connections using dormitory housing information and participants' class schedules for the Winter 2013 semester. Reported contacts in classrooms were further divided into small classes (<60 students) and large classes (⩾60 students). Students who reported being in the same class or who lived in the same room were considered to have contact over the entire study period in that location.

Alcohol consumption (drinker, non-drinker), hand hygiene practices (optimal, suboptimal), 2012–2013 influenza vaccination status (yes, no), gender (female, male) and class attendance behaviour while symptomatic (yes, no) were self-reported. Alcohol consumption was defined as drinking at least once a week. Optimal hand hygiene practice was defined as washing hands for at least 20 s at least five times a day. At study enrolment, intention to attend class while symptomatic and intention to attend class while symptomatic if an assignment or exam was due were collected.

### Analysis

We defined a transmission event as two students with positive PCR results for the same pathogen who had contact within 14 days before onset. We visualised each event through directed networks, where arrows indicate the direction of transmission. Directionality was determined by onset date. Transmitters were students who had an infection prior to their social contact and whose social contact became infected within 14 days. Infection recipients were students who were infected by their social contact (transmitter). One pair of infected students with the same onset date was not counted in the analyses because it was not possible to define either as a transmitter or a recipient (this pair is indicated in the network by a double-headed arrow). As this was a descriptive study, no statistical tests comparisons were performed. Uncertainty for proportions and means was expressed with Wald-type 95% confidence intervals. All analysis was performed using Python 3.5.2 (Python Software Foundation, Wilmington, DE, USA) and the NetworkX 2.1 library [22]. This study was approved by the US Centers for Disease Control and Prevention through institutional review board deferral to the University of Michigan and the University of North Carolina.

## Results

**Fig. 1.**
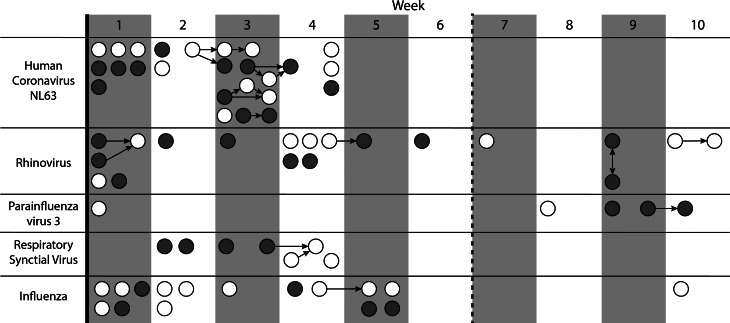
Transmission networks stratified by pathogen and incidence week. Students' onset dates had to be within 14 days of each other for their contact to be considered a potential transmission event. Double-headed arrows indicate the same onset date for connected nodes, so direction cannot be determined. Isolated nodes were cases with no identified transmission links. White nodes were randomised to the intervention arm and dark grey nodes were randomised to the control arm. A week-long spring break occurred between weeks 6 and 7, indicated by a dashed grey line. Median and IQR of days between onset by pathogen are as follows: influenza A (1 day), parainfluenza virus 3 (8 days), rhinovirus (2 days, IQR 1–2), human coronavirus NL63 (4 days, IQR 2–7), respiratory syncytial virus (5 days).

During the 10-week study period, 110 (19%) of 590 enrolled participants reported 132 influenza-like illness events. Of all positive samples (*n* = 104), the following pathogens were detected: HCoV-NL63 (25%), HCoV-229E (8%), HCoV-OC43 (5%), HCoV-HKU1 (3%), rhinovirus (18%), influenza A (15%), influenza B (4%), RSV (8%), hMPV (6%), parainfluenza virus 1 (1%), parainfluenza virus 3 (5%) and adenovirus (3%). Descriptive statistics for individuals with at least one positive sample for the listed pathogens are presented in Table 1. Putative virus transmission events (*n* = 18) were identified for influenza A (6%), parainfluenza virus 3 (6%), rhinovirus (22%), HCoV-NL63 (56%) and RSV (11%) (Fig. 1). The median onset time difference between pairs was 4 days (IQR 2–7 days).

**Table 1.** Descriptive characteristics of the eX-FLU study by positive viral pathogen test results

	At least one positive sample (*n* = 82)^a^		Total (*n* = 590)	
	*N* (%)/Mean	95% CL	*N* (%)/Mean	95% CL
Three-day isolation arm	39 (48%)	37–58%	287 (49%)	45–53%
Female	46 (56%)	45–67%	323 (58%)	54–62%
Missing	0		32	
Received 2012–2013 influenza vaccine	19 (25%)	15–35%	162 (40%)	35–45%
Missing	7		187	
Drink alcohol at least once a week	39 (51%)	39–62%	155 (37%)	32–41%
Missing	5		167	
Optimal hand hygiene^b^	20 (25%)	15–34%	126 (29%)	25–34%
Missing	1		162	
Attend class while symptomatic	28 (42%)	31–54%	40%	35–45%
Missing	16		275	
Attend class while symptomatic if exam/assignment due	58 (88%)	80–96%	274 (87%)	83–91%
Missing	16		275	
Number of social contacts^a^	15	14, 17	10	9, 11

a At least one positive sample over the study period for one of the following viral pathogens: human coronavirus (HCoV) NL63, HCoV-229E, HCoV-OC43, HCoV-HKU1, rhinovirus, influenza A, influenza B, respiratory syncytial virus, human metapneumovirus, parainfluenza virus 1/2/3 or adenovirus.

b Optimal hand hygiene is defined as self-report of washing hands at least five times per day for at least 20 s.

^c^Number of reported contacts a participant had over the entire study period. This reflects a person's position within the larger contact network.

Of transmitters (*n* = 15), less than half were in the control arm of the trial (0.47, 95% CL 0.21–0. 72), male (0.33, 95% CL 0.08–0.57), received the 2012–13 influenza vaccine (0.29, 95% CL 0.05–0.52), drank alcohol at least once a week (0.47, 95% CL 0.21–0.72) and reported optimal hand hygiene (0.40, 95% CL 0.15–0.65). Transmitters had a higher number of co-infections or secondary infections than the infection recipients, and more than half reported at least one respiratory symptom (Table 2). Two transmitters (0.13, 95% CL 0.0–0.31) reported no symptoms.

**Table 2.** Characteristics of students with positive samples for influenza A, parainfluenza virus 3, rhinovirus, human coronavirus NL63 or respiratory syncytial virus

		Transmitters		Infection recipients		Unlinked	
		(*n* = 15)		(*n* = 14)		(*n* = 39)	
		*N* (%)/Mean	95% CL	*N* (%)/Mean	95% CL	*N* (%)/Mean	95% CL
Three-day isolation arm		8 (53%)	28–79%	9 (64%)	39–89%	19 (49%)	33–64%
Female		10 (67%)	43–91%	6 (43%)	17–69%	21 (54%)	38–69%
Received 2012–2013 influenza vaccine		4 (29%)	5–52%	2 (15%)	4–35%	10 (29%)	14–44%
	Missing	1		1		4	
Drink alcohol at least once a week		7 (47%)	21–72%	10 (71%)	48–95%	18 (53%)	36–70%
	Missing	0		0		5	
Optimal hand hygiene^a^		6 (40%)	15–65%	2 (15%)	0–33%	9 (24%)	10–37%
	Missing	0		0		1	
Attend class while symptomatic		6 (43%)	17–69%	5 (42%)	14–70%	12 (40%)	22–58%
	Missing	1		2		9	
Attend class while symptomatic if exam/assignment due		12 (86%)	67–100%	11 (92%)	76–100%	26 (87%)	75–99%
	Missing	1		2		9	
Number of social contacts^b^		20	16–23	19	15–24	14	12–17
Co-infection/secondary infection							
	Single virus	5 (33%)	9–57%	10 (71%)	48–95%	18 (46%)	31–62%
	Multiple viruses	1 (7%)	0–19%	1 (7%)	0–21%	2 (5%)	0–12%
	Virus and bacteria	6 (40%)	15–65%	1 (7%)	0–21%	7 (18%)	6–30%
	Multiple viruses and bacteria	3 (20%)	0–40%	1 (7%)	0–21%	4 (10%)	1–20%
	Multiple bacteria and virus	0 (0%)	0–22%	1 (7%)	6–21%	3 (8%)	0–16%
Symptoms							
	Abdominal pain	2 (13%)	0–31%	0 (0%)	0–23%	6 (15%)	4–27%
	Body ache	6 (40%)	15–65%	3 (21%)	0–43%	17 (44%)	28–59%
	Chills	5 (33%)	9–57%	2 (14%)	0–33%	13 (32%)	19–48%
	Coughing	11 (73%)	51–96%	7 (50%)	24–76%	28 (70%)	58–86%
	Earache	2 (13%)	0–31%	0 (0%)	0–23%	6 (15%)	4–27%
	Fever	3 (20%)	0–40%	0 (0%)	0–23%	17 (44%)	28–59%
	Headache	9 (60%)	35–85%	3 (21%)	0–43%	22 (56%)	41–72%
	Nasal congestion	10 (67%)	43–91%	9 (64%)	39–89%	28 (70%)	58–86%
	Runny nose	10 (67%)	43–91%	7 (50%)	24–76%	31 (79%)	67–92%
	Sneezing	8 (53%)	28–79%	6 (43%)	17–69%	26 (67%)	52–81%
	Sore throat	11 (73%)	51–96%	8 (57%)	31–83%	27 (69%)	55–84%
	Respiratory symptoms^c^	13 (87%)	69–100%	11 (79%)	57–100%	33 (85%)	73–96%
	Multiple symptoms	13 (87%)	69–100%	11 (79%)	57–100%	33 (85%)	73–96%
	No symptoms	2 (13%)	0–31%	2 (14%)	0–33%	2 (5%)	2–12%

IQR, interquartile range. Transmitters were any students who were indicated as transmitting an infection to at least one social contact. Infection recipients were any students who were indicated as having the infection transmitted to them via at least one social contact. Unlinked cases were students who had no defined transmission events in either direction. Students were eligible to be counted once in each category.

a Optimal hand hygiene is defined as self-report of washing hands at least five times per day for at least 20 s.

b Number of reported contacts a participant had over the entire study period. This reflects a person's position within the larger contact network.

c Defined as the presence of at least one of the following symptoms: coughing, nasal congestion, runny nose or sneezing.

Of infection recipients (*n* = 14), less than half were in the control arm (0.36, 95% CL 0.11–0.61), female (0.43, 95% CL 0.17–0.69) and reported optimal hand hygiene (0.15, 95% CL 0.0–0.33). Over half of infection recipients did not receive the 2012–13 influenza vaccine (0.85, 95% CL 0.65–0.96) and drank alcohol at least once a week (0.71, 95% CL 0.36–0.70). Intended class attendance was similar between transmitters, recipients and unlinked participants (Table 2). Infection by a single virus was detected in 0.71 (95% CL 0.48–0.95) infection recipients, which was lower than transmitters (0.33, 95% CL 0.09–0.57). The most commonly reported symptoms were nasal congestion, sore throat, runny nose and coughing (Table 2). Symptoms were broadly more common in transmitters and unlinked participants relative to recipients. Two infection recipients (0.14, 95% CL 0–33%) reported no symptoms.

### Interaction context

Most of the identified transmission events occurred between friends (0.56, 95% CL 0.33–0.79), classmates (0.33, 95% CL 0.12–0.55), coworkers (0.17, 95% CL 0–34%) or study partners (0.17, 95% CL 0.0–0.34) (Table 3). Only two transmission events were identified between roommates (0.11, 95% CL 0.03–0.26). All transmission events with reported relationships (0.78, 95% CL 0.59–0.97) had multiple relationship types. Most transmission events were self-reported contacts indicated by both students (0.67, 95% CL 0.45–0.88), and students of transmission pairs reported contact multiple times over the 10-week study period (mean = 6, 95% CL 4–8). Half of the transmission events had no location indicated by either student. However, a majority of identified transmission events were between students who resided in the same dormitory (0.67, 95% CL 0.45–0.88). Of transmission events with reported locations, most commonly contact locations related to transmission occurred in dormitory common areas, dormitory rooms, small classes and cafeterias (Table 3). All transmission events had reported contact in multiple locations.

**Table 3.** Characteristics of identified transmission events

		Transmission events (*n* = 18)	
		*N* (%)/Mean	95% CL
Type of relation between contacts			
	Roommate	2 (11%)	3–26%
	Classmate	6 (33%)	12–55%
	Friend	10 (56%)	33–79%
	Study partner	3 (17%)	0–34%
	Teammate	2 (11%)	0–26%
	Romantic	1 (6%)	0–16%
	Family	1 (6%)	0–16%
	Coworker	3 (17%)	0–34%
	More than one	14 (78%)	59–97%
	None indicated	4 (22%)	3–41%
Self-reported contacts			
	Important contact^a^	6 (33%)	12–55%
	Times nominated over study	6	4–8
	Dual nominations	12 (67%)	45–88%
	Same dormitory	12 (67%)	45–88%
Location of contact			
	Dorm room	3 (17%)	0–34%
	Dorm common area	4 (22%)	3–41%
	Class	6 (33%)	12–55%
	Cafeteria	2 (11%)	0–26%
	Party	1 (6%)	0–16%
	Public transport	1 (6%)	0–16%
	Workplace	1 (6%)	0–16%
	Multiple locations	9 (50%)	27–73%
	None indicated	9 (50%)	27–73%

Transmission events correspond to a viral transmission between two students and were defined as two students with positive PCR results for the same pathogen who had contact within a 14-day period. Transmission event corresponds to the arrows in Figure 1.

a Contacts were considered to be important if either student selected the other as one of the top three people they had contact with during that week.

## Discussion

In this study, we identified putative transmission events for five respiratory viruses by integrating molecular methods with a network of students' social contacts at a large university. Our descriptive analysis highlights that both biological factors and social factors need to be considered in transmission studies. Regarding biological factors, the occurrence of co-infections and respiratory symptoms capable of aerosolisation, such as coughing and sneezing, was observed more frequently in transmitters. Interestingly, 13% of transmitters reported no symptoms. The extent of asymptomatic transmitters identified is similar to the overall incidence of asymptomatic cases [21]; and to previous reports of the overall incidence of asymptomatic cases of rhinovirus among university students [15] and pandemic 2009 influenza H1N1 at schools in India and China [23, 24]. Regarding social factors, females constituted a majority of transmitters, while infection recipients were a majority of males. Females have been previously observed to have larger social networks [25], and this observation held in our data (Supplementary Table S1). The greater extent of contacts may partially explain increased identification of females as sources of infection. Infection recipients reported lower optimal hand hygiene and influenza vaccination. However, reported intention to attend class while ill was similar between transmitters and recipients. Finally, transmission events largely occurred in dormitories between close contacts but we also found evidence of transmission in smaller classes.

Our results expand on previous findings of respiratory illness transmission among university students [6–8, 10–12, 16]. Transmission events have been previously identified between close contacts. Previous research has suggested studying with an ill individual [12], living/sharing a room with an ill individual [11, 12, 16] or caring for an ill individual [12] as risk factors for respiratory transmission. Similarly, transmission events in our study occurred between contacts with multiple relationship categories and in multiple locations. These results suggest transmission events were limited to close contacts between individuals. This conclusion is further supported by the fact that the majority of transmission events occurred between students who lived in the same dormitory residence hall, and identified transmission pairs reported continued contact with each other over follow-up. A substantive number of contacts had no indicated relationship type or location. However, it is difficult to discern if this is missing data or indicating the absence of a relationship. Putative transmission events were observed in smaller. Prior research has found no evidence of transmission occurring in classes of any size. The size of classes may play an important role, with additional social pressure to attend class while ill may occur in smaller classes due to absences being more readily noticed compared to larger classes. Potentially the context of interactions between classmates may differ based on class sizes. The extent that classrooms, particularly by class size, contribute to respiratory infection transmission should be explored in future studies.

While the influenza vaccine is the primary preventative measure for seasonal influenza, other seasonal respiratory pathogens and emerging infections require the use of non-pharmaceutical interventions to interrupt transmission, with the SARS-CoV-2 pandemic serving as a stark reminder. With regards to seasonal transmission, previous work has indicated improved hand hygiene and the use of face masks can reduce transmission among college students [26–28]. Furthermore, social distancing is a promising intervention to reduce transmission but may be difficult to introduce in the university setting due to the population density of dormitories and shared rooms. These interventions are similarly expected to be beneficial during respiratory infection pandemics. Institution of these measures by universities may be required, but some evidence exists that university students reasonably adhere to those non-pharmaceutical interventions recommendations during epidemics [29]. However, staying home while symptomatic has been previously observed to be low and may require a robust response from universities to encourage this behaviour.

The major limitation of our study is that many infected students were not linked. Several explanations are possible: underreporting of contacts, undetected asymptomatic spreaders or introductions of the pathogen from outside the observed network. It has been previously observed that self-reporting underrepresents the true number of contacts [30–34]. The observation that transmission events occurred between students with multiple relations and locations may be explained by the differential recall of close contacts *vs.* transient contacts [30]. However, we supplemented self-reported data with information regarding class schedules and housing information. Asymptomatic transmitters may be underrepresented, since the nasal sampling procedure selected symptomatic individuals or individuals who reported contact with symptomatic individuals. However, previous studies in university settings have had no mechanism with which to detect transmission by asymptomatic individuals. Next, the presented confidence intervals implicitly assume observations are independent, which is untrue in network data. Therefore, the presented confidence intervals indicate less uncertainty than necessary. Additionally, we do not have a census of the social contact network. As a result, individuals not enrolled in the study may be the missing links between observed transmission events. Transmission direction has the potential to be misspecified because incubation periods differ between individuals or because some pathogens are contagious prior to symptom onset (e.g. influenza). Furthermore, we are unable to separate true transmission events from concurrent infections acquired from a common source. Next, relatively few transmission events occurred for each pathogen, and because all pathogens were respiratory viruses, results were collapsed together. Lastly, immunity at baseline was not captured and a lack of immunity may explain the substantial burden of CoV-NL63 transmission observed in this population.

## Conclusions

This study used a novel design with both ego and alter reported contacts to capture both influenza and other respiratory infection transmission events. By integrating social networks and molecular typing of organisms, we identified transmission events for a variety of respiratory pathogens among university students – an understudied population for respiratory infections. Under 20% of influenza-like illness cases tested positive for influenza, reflecting that influenza-like illness symptoms are non-specific to influenza and suggesting studies should not rely solely on influenza-like illness to identify transmission events in this age group. Observed transmission chains occurred between friends or classmates, and contacts occurred in dormitories or small classes. The occurrence of transmission in smaller classes deserves future exploration on what drives transmission in those settings. Factors that may foster greater aerosol and possibly even contact transmission in small classes include a small room size, proximity of seating and extent of student discussion. Overall, our results support the use of interventions that target aerosolisation, like face masks and isolation, to prevent transmission. However, strategies that target asymptomatic infections or fomites also need to be considered. Indeed, prior research has suggested that interventions may need to interrupt transmission along multiple routes simultaneously to be successful. Future work should continue the integration of molecular methods, well-characterised social network analysis and traditional epidemiology to further understand transmission dynamics, modes of transmission and ultimately how to mitigate the spread of disease. In particular, these approaches will be integral for tracking and identifying the transmission of SAR-CoV-2 in human populations.

## Supplementary material

For supplementary material accompanying this paper visit http://dx.doi.org/10.1017/S0950268820001806. click here to view supplementary material
