# Supplementary material for: Transmission of viral pathogens in a social network of university students: the eX-FLU study
Source: Epidemiol Infect. 2020 Aug 14;148:e267. doi: 10.1017/S0950268820001806 (PMC7689784; doi:10.1017/S0950268820001806)
Supplement: Supplementary file 1 [file S0950268820001806sup001.docx]

**Transmission of viral pathogens in a social network of university students: The eX-FLU Study**

Paul N. Zivich, Marisa C. Eisenberg, Arnold S. Monto, Amra Uzicanin, Ralph S. Baric, Timothy P. Sheahan, Jeanette J. Rainey, Hongjiang Gao, Allison E. Aiello

Below are results presented for the full social contact network study. The number of contacts (degree) within the network is reported stratified by week of follow-up and by self-reported gender.

| Supplementary Table 1. Number of Unique Contacts by Week and Self-reported Gender | | |
| --- | --- | --- |
|  | Mean (SD) number of unique contacts | |
|  | Female (n=323) | Male (n=235) |
| Week 1 | 8.3 (7.3) | 6.8 (6.3) |
| Week 2 | 8.6 (7.6) | 7.2 (6.9) |
| Week 3 | 8.5 (7.8) | 7.2 (7.3) |
| Week 4 | 8.5 (7.7) | 7.2 (7.5) |
| Week 5 | 8.4 (7.8) | 7.2 (7.3) |
| Week 6 | 7.9 (7.4) | 6.7 (7.2) |
| Week 7 | 8.0 (7.7) | 6.8 (7.3) |
| Week 8 | 8.1 (7.6) | 6.7 (7.1) |
| Week 9 | 8.0 (7.6) | 6.5 (6.9) |
| Week 10 | 8.0 (7.6) | 6.9 (7.4) |
| SD: standard deviation. Gender was not reported for 32 individuals. | | |
